# Supplementary material for: Association of egg consumption, metabolic markers, and risk of cardiovascular diseases: A nested case-control study
Source: eLife. 2022 May 24;11:e72909. doi: 10.7554/eLife.72909 (PMC9129873; doi:10.7554/eLife.72909)
Supplement: Transparent reporting form [file elife-72909-transrepform1.docx]

***eLife’s* transparent reporting form**

We encourage authors to provide detailed information *within their submission* to facilitate the interpretation and replication of experiments. Authors can upload supporting documentation to indicate the use of appropriate reporting guidelines for health-related research (see [EQUATOR Network](http://www.equator-network.org/%20)), life science research (see the [BioSharing Information Resource](https://biosharing.org/)), or the [ARRIVE guidelines](http://www.plosbiology.org/article/info:doi/10.1371/journal.pbio.1000412) for reporting work involving animal research. Where applicable, authors should refer to any relevant reporting standards documents in this form.

If you have any questions, please consult our Journal Policies and/or contact us: [editorial@elifesciences.org](mailto:editorial@elifesciences.org).

**Sample-size estimation**

- You should state whether an appropriate sample size was computed when the study was being designed
- You should state the statistical method of sample size computation and any required assumptions
- If no explicit power analysis was used, you should describe how you decided what sample (replicate) size (number) to use

Please outline where this information can be found within the submission (e.g., sections or figure legends), or explain why this information doesn’t apply to your submission:

The present analysis is based on an established population-based cohort study, CKB. We included as many eligible individuals as possible and didn’t calculate the sample size. Information on inclusion and exclusion criteria can be found in the subsection of ‘Participants and study design’ under ‘Methods’ part.

**Replicates**

- You should report how often each experiment was performed
- You should include a definition of biological versus technical replication
- The data obtained should be provided and sufficient information should be provided to indicate the number of independent biological and/or technical replicates
- If you encountered any outliers, you should describe how these were handled
- Criteria for exclusion/inclusion of data should be clearly stated
- High-throughput sequence data should be uploaded before submission, with a private link for reviewers provided (these are available from both GEO and ArrayExpress)

Please outline where this information can be found within the submission (e.g., sections or figure legends), or explain why this information doesn’t apply to your submission:

This informatiton, such as retreatment of metabolomics data and the processing of missing values, can be found in the subsection of ‘Statistical analysis’ under ‘Methods’ part.

**Statistical reporting**

- Statistical analysis methods should be described and justified
- Raw data should be presented in figures whenever informative to do so (typically when N per group is less than 10)
- For each experiment, you should identify the statistical tests used, exact values of N, definitions of center, methods of multiple test correction, and dispersion and precision measures (e.g., mean, median, SD, SEM, confidence intervals; and, for the major substantive results, a measure of effect size (e.g., Pearson's r, Cohen's d)
- Report exact p-values wherever possible alongside the summary statistics and 95% confidence intervals. These should be reported for all key questions and not only when the p-value is less than 0.05.

Please outline where this information can be found within the submission (e.g., sections or figure legends), or explain why this information doesn’t apply to your submission:

This informatiton can be found in the subsection of ‘Statistical analysis’ under ‘Methods’ part.

(For large datasets, or papers with a very large number of statistical tests, you may upload a single table file with tests, Ns, etc., with reference to sections in the manuscript.)

**Group allocation**

- Indicate how samples were allocated into experimental groups (in the case of clinical studies, please specify allocation to treatment method); if randomization was used, please also state if restricted randomization was applied
- Indicate if masking was used during group allocation, data collection and/or data analysis

Please outline where this information can be found within the submission (e.g., sections or figure legends), or explain why this information doesn’t apply to your submission:

This is an observational study, and allocation is not applicable.

**Additional data files (“source data”)**

- We encourage you to upload relevant additional data files, such as numerical data that are represented as a graph in a figure, or as a summary table
- Where provided, these should be in the most useful format, and they can be uploaded as “Source data” files linked to a main figure or table
- Include model definition files including the full list of parameters used
- Include code used for data analysis (e.g., R, MatLab)
- Avoid stating that data files are “available upon request”

Please indicate the figures or tables for which source data files have been provided:

For researchers who are interested to access the original data, the access policy and procedures are available at www.ckbiobank.org. In brief, the China Kadoorie Biobank (CKB) is being conducted jointly by the Clinical Trial Service Unit (CTSU), Nuffield Department of Population Health, University of Oxford, and Chinese Academy of Medical Sciences (CAMS) in Beijing. Requesters should be employees of a recognized academic institution, health service organization, or charitable research organization with experience in medical research. Requestors should be able to demonstrate, through their peer-reviewed publications in the area of interest, their ability to carry out the proposed study. After registration, details of the required information are provided on the CKB Data Access System. The CKB Access Team will review and respond to data requests within 6-8 weeks.

In order to explain more clearly, all the variables required for the analysis of this study are as follows:

1. Background: Basic [Baseline, 1st resurvey, 2nd resurvey], Demographics [Baseline];

2. Tea consumption: Basic [Baseline], Details [Baseline];

3. Alcohol consumption: Basic [Baseline], Details [Baseline];

4. Smoking: Basic [Baseline], Details [Baseline];

5. Diet: Staple foods [Baseline, 1st resurvey, 2nd resurvey], Animal products [Baseline, 1st resurvey, 2nd resurvey], Vegetables [Baseline, 1st resurvey, 2nd resurvey], Other foods [Baseline, 1st resurvey, 2nd resurvey], Drinks [2nd resurvey];

6. Medical history: Self-rated [Baseline], Personal [Baseline], Family [Baseline];

7. Physical activity: Summary - MET [Baseline];

8. Mental health: Satisfaction [Baseline];

9. Physical exam: Height and weight [Baseline], Body composition [Baseline], Blood pressure [Baseline], Blood glucose [Baseline];

10. Biochemistry data: Blood biomarkers (lab) [Baseline], Blood biomarkers (NMR) [Baseline], Blood biomarkers (NMR) - Chylomicrons and extremely large VLDL [Baseline], Blood biomarkers (NMR) - Very large VLDL [Baseline], Blood biomarkers (NMR) - Large VLDL [Baseline], Blood biomarkers (NMR) - Medium VLDL [Baseline], Blood biomarkers (NMR) - Small VLDL [Baseline], Blood biomarkers (NMR) - Very small VLDL [Baseline], Blood biomarkers (NMR) - IDL [Baseline], Blood biomarkers (NMR) - Large LDL [Baseline], Blood biomarkers (NMR) - Medium LDL [Baseline], Blood biomarkers (NMR) - Small LDL [Baseline], Blood biomarkers (NMR) - Very large HDL [Baseline], Blood biomarkers (NMR) - Large HDL [Baseline], Blood biomarkers (NMR) - Medium HDL [Baseline], Blood biomarkers (NMR) - Small HDL [Baseline], Blood biomarkers (NMR) - LDL [Baseline], Blood biomarkers (NMR) - HDL [Baseline], Blood biomarkers (NMR) - fatty acids [Baseline].

We uploaded Stata code that was used to analyze the data.

The numbers used to generate Figures 1 and 2 were actually table S1 and S2, which were also uploaded as Excel files. We also uploaded high-resolution raw images (TIF format) of Figures 1 and 2.
